# 肺癌患者术前呼气峰流速可以预测肺叶切除术后肺部并发症吗？

**DOI:** 10.3779/j.issn.1009-3419.2017.09.03

**Published:** 2017-09-20

**Authors:** 坤 周, 砚铭 吴, 建华 苏, 玉田 赖, 诚 沈, 鹏飞 李, 国卫 车

**Affiliations:** 1 610041 成都，四川大学华西医院胸外科 Department of Thoracic Surgery, West China Hospital, Sichuan University, Chengdu 610041, China; 2 610041 成都，四川大学华西医院胸康复科 Department of Rehabilitation, West China Hospital, Sichuan University, Chengdu 610041, China

**Keywords:** 术前呼气峰流速, 术后肺部并发症, 肺肿瘤, 肺外科手术, Peak expiratory flow, Postoperative pulmonary complications, Lung neoplasms, Pulmonary surgical srocedures

## Abstract

**背景与目的:**

术后肺部并发症（postoperative pulmonary complications, PPCs）尤其是术后肺炎（postoperative pneumonia, POP）的发生，直接影响肺癌患者术后的快速恢复。呼气峰流速（peak expiratory flow, PEF）反映气道通畅性和咳嗽效率，咳嗽能力不足可能和术后肺部并发症有关。本研究旨在探讨术前PEF能否预测肺癌患者术后肺部并发症。

**方法:**

回顾性分析2014年1月-2015年12月四川大学华西医院胸外科单个医疗组433例肺癌手术治疗的患者，分析术前PEF及术后肺部感染、肺不张、肺持续性漏气等肺部并发症，并记录相关临床资料。

**结果:**

术前PEF值在并发症组（280.93±88.99）L/min显著低于无并发症组（358.38±93.69）L/min（*P* < 0.001）；*Logistic*回归分析显示术前PEF值和手术时间是PPCs的独立危险因素；术前PEF阈值为320 L/min是预测PPCs发生的最佳临界值（AUC=0.706, 95%CI: 0.661-0.749），肺部并发症发生率PEF≤320 L/min组（26.6%）显著高于PEF > 320 L/min组（9.4%）（*P* < 0.001）。

**结论:**

肺癌患者术前PEF值和术后PPCs发生有一定相关性，有可能作为预测PPCs发生的指标。

## Abstract (en)

**Background and objective:**

Postoperative pulmonary complications (PPCs), especially postoperative pneumonia (POP), directly affect the rapid recovery of lung cancer patients after surgery. Peak expiratory flow (PEF) can reflect airway patency and cough efficiency. Moreover, cough impairment may lead to accumulation of pulmonary secretions which can increase the risk of PPCs. The aim of this study is to investigate the effect of preoperative PEF on PPCs in patients with lung cancer.

**Methods:**

Retrospective research was conducted on 433 lung cancer patients who underwent lobectomy at the West China Hospital of Sichuan University from January 2014 to December 2015. The associations between preoperative PEF and PPCs were analyzed based on patients' basic characteristics and clinical data in hospital.

**Results:**

Preoperative PEF value in PPCs group (280.93±88.99) L/min was significantly lower than that in non-PPCs group (358.38±93.69) L/min (*P* < 0.001). According to the binary logistics regression analysis, PEF and operative time were independent risk factors for PPCs. Further, ROC curve showed that PEF=320 L/min was the cut-off value for predicting the occurrence of PPCs (AUC=0.706, 95%CI: 0.661-0.749). The incidence of PPCs in PEF≤320 L/min group (26.6%) was significantly higher than that in PEF > 320 L/min group (9.4%)(*P* < 0.001).

**Conclusion:**

Preoperative PEF and PPCs are correlated, and PEF may be used as a predictor of PPCs.

外科手术仍是肺癌患者治疗的主要手段，而术后肺部并发症（postoperative pulmonary complications, PPCs），尤其是术后肺炎（postoperative pneumonia, POP），不但延长了患者住院时间，同时也是患者治疗失败和术后死亡的主要原因^[1, 2]^。因此，降低术后肺部并发症不但能够加速术后康复，也有助于提高患者满意度和降低医疗费用^[3]^，然而目前常用的肺功能指标，如一秒用力呼气容积（forced expiratory volume in one second, FEV_1_）不足以准确预测或评估术后肺部并发症的发生^[4]^。呼气峰流速（peak expiratory flow, PEF）是反映气道通畅性和呼吸肌力量的一个敏感指标^[5]^，可以衡量咳嗽咳痰的效率。肺癌患者术后咳嗽能力降低会引发痰潴留进而导致肺部感染、肺不张等并发症的发生。我们研究发现术前短期肺康复训练，可以有效提高PEF值^[6, 7]^。而术前PEF值与PPCs发生相关性的临床研究尚无报道。本研究旨在探索术前PEF值和PPCs的相关性，评价其在预测评估PPCs发生上的应用价值，为加速康复外科（enhanced recovery after surgery, ERAS）在围术期的流程优化提供证据和策略^[8]^。

## 1 资料和方法

### 1.1 研究对象

回顾性分析华西医院胸外科2014年1月-2015年12月单个医疗组535例肺癌患者的临床资料。纳入标准：①术后病理诊断为原发性非小细胞肺癌；②手术方式为肺叶除术+系统淋巴结清扫术；③术前未应用抗生素治疗；④临床病历资料完整者。排除标准：①术前存在明确的肺部感染或心、脑、肾相关疾病；②全肺切除或行支气管袖式成形肺叶切除术；③术前接受放化疗者；④临床病历资料缺失者。最终排除患者102例，纳入患者433例。根据是否发生肺部并发症，将其分为两组，其中并发症（PPCs）组75例，无并发症（Non-PPCs）组358例。术后分期采用国际抗癌联盟（Union for International Cancer Control, UICC）^[9]^（第七版）肺癌分期标准，患者临床特征见表 1。

**1:** 两组患者临床特征 The characteristics of the patients

Characteristics	PPCs group (*n*=75)	Non-PPCs group (*n*=358)	*P*
Age (Mean±SD, yr)	64.95±8.91	60.70±9.27	< 0.001
BMI (Mean±SD, kg/m^2^)	23.19±3.25	23.46±2.92	0.484
PEF (Mean±SD, L/min)	280.93±88.99	358.38±93.69	< 0.001
Gender			0.058
Male	51 (68.0%)	201 (56.1%)	
Female	24 (32.0%)	157 (43.9%)	
Smoking status			0.003
Current/ever	43 (57.3%)	139 (38.8%)	
Never	32 (42.7%)	219 (61.2%)	
Comorbidities			
Diabetes	9 (12.0%)	34 (9.5%)	0.510
Hypertension	22 (29.3%)	94 (26.3%)	0.584
COPD	24 (32.0%)	47 (13.1%)	< 0.001
Surgical approach			0.001
Open	32 (42.7%)	87 (24.3%)	
VATS	43 (57.3%)	271 (75.7%)	
Histology			0.289
Adenocarcinoma	36 (48.0%)	188 (52.5%)	
Squamous carcinoma	27 (36.0%)	129 (36.0%)	
Other	12 (16.0%)	41 (11.5%)	
TNM stage			0.428
Stage Ⅰ	39 (52.0%)	205 (57.3%)	
Stage Ⅱ	24 (32.0%)	102 (28.5%)	
Stage Ⅲ+Ⅳ	12 (16.0%)	51 (14.2%)	
PEF: peak expiratory flow; COPD: chronic obstructive pulmonary disease; VATS: video-assisted thoracic surgery; PPC: postoperative pulmonary complications.			

### 1.2 方法

手术方式应用开胸或单向式胸腔镜肺叶切除+系统淋巴结清扫，左侧必须清扫第5、6、7、8、9、10组淋巴结，右侧包括第2、3、4、7、8、9、10组淋巴结^[10]^。术后两组患者均采用静脉自控镇痛泵（patient controlled intravenous analgesia, PCIA）为基础镇痛方式，镇痛泵内药物为芬太尼、曲马多、托烷司琼和0.9%生理盐水配至100 mL，持续剂量2 mL/h。

### 1.3 肺部并发症

肺部并发症^[11, 12]^包括：（1）肺部感染：术后胸片出现新发渗出，实变或空洞影，同时满足以下一项或更多条件：①出现脓痰或者有病原学证据；②发热 > 38 ℃；③白细胞数 < 4 ×10^9^/或 > 12×10^9^/L；④术后更换抗生素类别或延长使用时间。（2）肺不张：肺组织局部密度增高，纵膈或肺门向病变区域移位，邻近正常肺组织代偿性肺气肿。（3）持续肺漏气：时间 > 7天并需要临床干预。（4）胸腔积气：胸片提示胸腔积气 > 30%，并再次置管。（5）重度皮下气肿：患者手术切口同侧和对侧胸壁、头面颈部出现皮下气肿。（6）胸腔积液：胸片提示积液中到大量。（7）支气管痉挛：术后出现呼气喘鸣并使用支气管扩张药物治疗。（8）呼吸衰竭或急性呼吸窘迫综合征（acute respiratory distress syndrome, ARDS）：术后持续通气支持 > 24 h或需要重新插管。（9）支气管胸膜瘘：经纤支镜证实。

### 1.4 研究指标

① 术前PEF：术前一天床旁应用峰值流速仪进行检测，患者吹三次，取最大值。②手术时间：从麻醉开始到患者拔除气管插管后回到复苏室。③术后抗生素使用时间：手术当日到停用抗生素。④术后住院日：手术当天到出院当天时间（出院当天计算在内，实际上应去除）。⑤住院总费用：住院期间所产生的费用，不包括门诊检查或治疗所产生的费用。

### 1.5 统计学方法

应用SPSS 23.0统计软件包进行分析。正态分布计量资料以均数±标准差（Mean±SD）表示，比较采用两独立样本*t*检验。计数资料采用实例数和百分比表示，比较采用χ^2^检验或*Fisher*确切概率法。多因素分析采用二分类*Logistic*回归，统计检验均采用双侧检验，*P* < 0.05为差异有统计学意义。

## 2 结果

### 2.1 两组患者（PPCs组和Non-PPCs组）临床特征分析

患者平均年龄在PPCs组（64.95±8.91）岁显著高于Non-PPCs组（60.70±9.27）岁（*P* < 0.001）；术前PEF值在PPC组（280.93±88.99）L/min显著低于Non-PPCs组（358.38±93.69）L/min（*P* < 0.001）；PPCs组患者在吸烟史（57.3% *vs* 38.8%, *P*=0.003）、合并慢性阻塞性肺病（chronic obstructive pulmonary disease, COPD）（32.0% *vs* 13.1%, *P* < 0.001）、开胸比例（42.7% *vs* 24.3%, *P*=0.001）均显著高于Non-PPCs组（表 1）。而两组患者在性别、BMI、糖尿病、高血压病、病理类型以及肿瘤分期上均无统计学差异。

### 2.2 术后PPCs危险因素分析

采用*Logistic*进行多因素回归分析，结果显示：PEF（OR=0.989, 95%CI: 0.985-0.993, *P* < 0.001）和手术时间（OR=1.016, 95%CI: 1.010-1.023, *P* < 0.001）是PPCs的独立危险因素（表 2）。

**2:** 术后肺部并发症危险因素分析 The *Logistic* analysis of risk factors for postoperative pulmonary complications

Variables	OR	95% confidence interval		*P*
		Lower bound	Upper bound	
Age	1.035	0.998	1.074	0.063
Gender	2.286	0.857	6.097	0.099
PEF	0.989	0.985	0.993	< 0.001
Smoking	1.526	0.641	3.637	0.340
COPD	1.618	0.819	3.197	0.166
Surgical approach	1.818	0.978	3.378	0.059
Operation time	1.016	1.010	1.023	< 0.001

### 2.3 术前PEF值预测PPCs发生的阈值

使用ROC曲线分析PEF对PPCs的预测价值，结果显示PEF预测PPCs发生的最佳阈值为320 L/min，敏感度70.67%，特异度59.22%，曲线下面积（area under the ROC curve, AUC）为0.706，95%CI: 0.661-0.749（图 1）。

**1:**
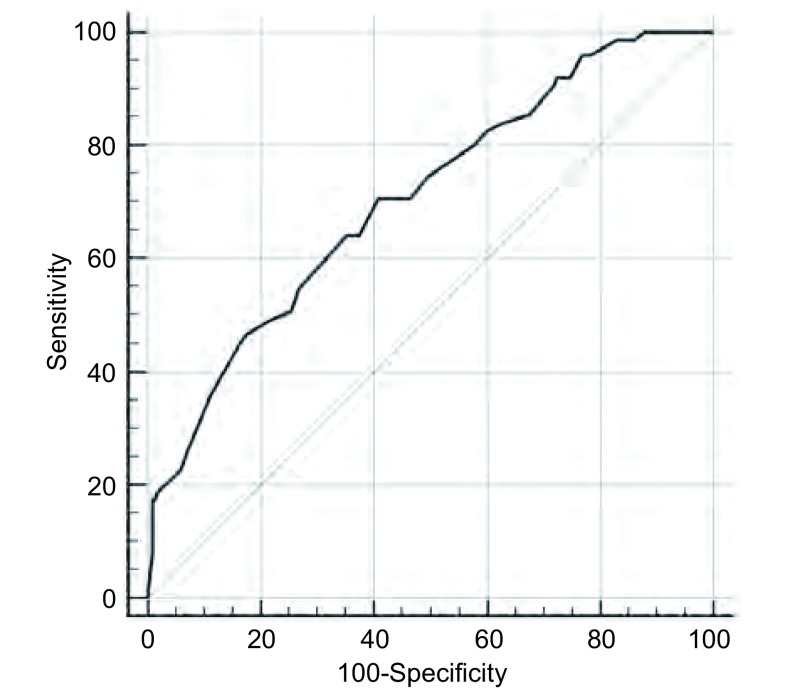
呼气峰流速预测术后肺部并发症的ROC曲线 ROC curve of postoperative pulmonary complications predicted by peak expiratory flow

### 2.4 PEF阈值上下两组患者临床特征比较

以术前PEF值320 L/min为阈值，199例肺癌患者术前PEF≤320 L/min，234例PEF > 320 L/min。分析发现肺部并发症发生率在PEF≤320 L/min组（26.6%）显著高于PEF > 320 L/min组（9.4%）（*P* < 0.001）。术后抗生素使用天数、住院总费用和术后住院日在PEF≤320 L/min组[(4.94±3.31) d, (8, 957.76±3, 682.63) ¥, (7.32±4.35) d]均显著高于PEF > 320 L/min组[(4.19±2.76) d, (7, 866.45±2, 776.82) ¥, (6.30±2.87) d]（*P*=0.012, *P*=0.005, *P*=0.002）（表 3）。

**3:** PEF阈值分组患者临床特征 The characteristics of two groups of patients who are demarcated by PEF cut-off value

Characteristics	PEF≤320 L/min (*n*=199)	PEF > 320 L/min (*n*=234)	*P* value
Age (yr)	63.42±8.64	59.75±9.59	< 0.001
BMI (kg/m^2^)	23.27±3.25	23.46±2.92	0.364
PEF (L/min)	257.19±48.01	419.62±80.31	< 0.001
Gender			< 0.001
Male	79 (39.7%)	173 (73.9%)	
Female	120 (60.3%)	61 (26.1%)	
Comorbidities			
Diabetes	23 (11.6%)	20 (8.5%)	0.296
Hypertension	58 (29.1%)	58 (24.8%)	0.307
COPD	44 (22.1%)	27 (11.5%)	0.003
Smoking status			
Current/ever	68 (34.2%)	114 (48.7%)	0.002
Never	131 (65.8%)	120 (51.3%)	
Surgical approach			0.173
Open	61 (30.7%)	58 (24.8%)	
VATS	138 (69.3%)	176 (75.2%)	
PPCs rate	53 (26.6%)	22 (9.4%)	< 0.001
Pulmonary infection	43 (21.6%)	15(6.4%)	< 0.001
Atelectasis	16 (8.0%)	7 (3.0%)	0.020
Air leak > 7 d	12 (6.0%)	9 (3.8%)	0.292
Pneumothorax	6 (3.0%)	6 (2.6%)	0.776
Subcutaneous emphysema	6 (3.0%)	5 (2.1%)	0.614
Pleural effusion	3 (1.5%)	3 (1.3%)	1.000
Bronchospasm	4 (2.0%)	1 (0.4%)	0.185
Respiratory failure or ADRS	1 (0.5%)	0 (0.0%)	0.460
Bronchopleural fistula	0 (0.0%)	1 (0.0%)	1.000
Operation time (Mean±SD, min)	123.22±47.19	119.97±47.30	0.507
Amount of blood loss (Mean±SD, mL)	86.43±46.52	92.89±42.17	0.131
Antibiotic use time (Mean±SD, d)	4.94±3.31	4.19±2.76	0.012
Postoperative hospital stay (Mean±SD, d)	7.32±4.35	6.30±2.87	0.005
Drug cost (Mean±SD, ¥)	8, 952.76±3, 682.63	7, 866.45±2, 776.82	0.001
Total cost (Mean±SD, ¥)	48, 602.81±12, 080.50	47, 502.02±10, 675.60	0.315
BMI: body mass index; ADRS: acute respiratory distress syndrome.			

## 3 讨论

肺癌患者术后肺部并发症是影响其术后康复的主要因素，降低和预防术后肺部并发症是快速肺康复的基础。因此，加速康复外科围术期流程优化的第一步，就是针对围术期危险因素的准确评估和合理干预^[8]^。目前术后肺部并发症没有统一的定义和标准，多数临床研究由于纳入排除标准的差异、种族差别、临床问题的处理方式不同，在进行PPCs危险因素分析时差异较大，但吸烟史、COPD、较差的肺功能状态是众多研究中较认可的相关危险因素^[4, 12, 13]^。其中肺功能检测是肺癌患者术前较为常用和客观的检查，已有多个研究分析了肺功能检测对肺手术风险的评估价值^[14]^，而肺功能指标PEF与PPCs的关系尚不明确。PEF是爆发性呼气，和咳嗽、呵气动作有相同的机制，即腹肌和膈肌快速收缩使腹内压增高，由于腹腔内容物几乎不能压缩，随之膈肌上抬同时胸廓收缩使胸腔容量减小，最后形成高压气体快速呼出^[15, 16]^，目前多用于呼吸肌力量和支气管哮喘的动态随访，而在肺外科领域的应用相对较少。影响PEF的因素包括以下几个方面：①胸廓结构的完整和呼吸肌功能的健全；②气管-支气管的通畅程度；③肺实质结构的健全和正常的弹性。咳嗽和呵气是清除气道分泌物的有效方式，同时也有助于肺切除术后胸腔积气积液的排出。随着胸腔镜的普及，肺癌患者的手术适应症不断扩大，对肺功能的要求不断降低^[17]^，然而问题是常常手术顺利完成，患者却因为难以自行排出痰液、积气、积液而导致肺部并发症。目前护理宣教、震动拍背、气管压迫辅助咳痰、镇痛祛痰药物的使用使得肺癌患者术后肺部并发症的发生率有所降低，但肺部感染、肺不张仍是肺癌患者术后最常见的肺部并发症，国外相关研究显示术后肺部感染的发生率在6%-25%^[18-20]^，同时也是肺切除术后发生率最高的肺部并发症。发生肺部感染的原因之一可能是部分肺癌患者呼吸肌力量较弱，咳嗽咳痰效率差，直观的表现就是PEF较低，加之40%-70%的肺癌患者合并COPD^[21]^，气道炎性介质作用和手术麻醉药物、气管插管的刺激使痰液生成增多，痰液排除障碍和生成增多导致痰潴留^[22]^，增加了肺部感染的几率。

从我们的研究结果可以看出PPCs组PEF平均值明显低于Non-PPCs组，进行*Logistic*多因素回归分析后，结果表明PEF、手术时间是PPCs的独立危险因素，进一步验证了PEF在预测评估PPC发生的有效性。通过ROC曲线计算得出PEF预测PPCs发生的最佳阈值为320 L/min，即PEF≤320 L/min时，提示术后发生PPCs的可能性较大。以阈值为分界点对患者分组后，我们可以看到PEF≤320 L/min组肺部并发症发病率明显高于PEF > 320 L/min组（26.6% *vs* 9.4%, *P* < 0.001），其中肺部感染和肺不张发生率有统计学差异，同时抗生素使用天数、术后住院日和住院药费也均显著高于PEF > 320 L/min组。基于这些结果，我们认为当PEF≤320 L/min时，可对患者进行适当的术前干预，这为规范和统一肺康复方案提供了循证学证据，并对肺癌患者术前康复锻炼的指征进行了一定的补充^[23]^。Hiroshi通过超声探测，发现PEF和腹外斜肌的厚度相关，而与腹直肌、腹内斜肌、腹横肌没有相关性^[15]^；赖玉田研究表明，术前短期药物康复联合呼吸训练、下肢耐力训练可以明显提高PEF值^[24]^，表明针对性的合理干预可以有效提高PEF值，这对PEF进一步的临床干预策略具有指导意义。PEF可用简易峰速仪检测，使用成本低，操作简单、便捷，患者在床旁即可检测，易于临床动态观察^[25]^，同时PEF还可用于评估肺康复锻炼效果，不同级别的医院均可适用。

本研究还存在一定的不足，由于是回顾性分析研究，存在病历资料记录失真和回忆偏倚；目前肺部并发症还没有统一的标准，作为单中心的回顾性分析，其结果的普遍性和推广性难免受到限制；虽然本研究明确了PEF对肺癌患者术前风险评估的价值，但仍需进一步的前瞻性、大样本、多中心研究加以论证。

总之，PEF检测是一项成本低，可重复，易操作的检查，可用于肺癌患者术前风险评估，对术后肺部并发症的发生有一定的预测作用，具有临床推广应用价值。
